# Load matters: neural correlates of verbal working memory in children with autism spectrum disorder

**DOI:** 10.1186/s11689-018-9236-y

**Published:** 2018-06-01

**Authors:** Vanessa M. Vogan, Kaitlyn E. Francis, Benjamin R. Morgan, Mary Lou Smith, Margot J. Taylor

**Affiliations:** 1 Diagnostic Imaging & Research Institute, Hospital for Sick Children, 555 University Avenue, Toronto, Ontario M5G 1X8 Canada; 2 Department of Applied Psychology and Human Development, Ontario Institute for Studies in Education, University of Toronto, 252 Bloor Street West, Toronto, Ontario M56 1V6 Canada; 3 Department of Psychology, University of Toronto, 100 St. George St., Toronto, Ontario M5S 3G3 Canada

**Keywords:** Autism spectrum disorder, Verbal working memory, Cognitive load, Executive functioning, fMRI

## Abstract

**Background:**

Autism spectrum disorder (ASD) is a pervasive neurodevelopmental disorder characterised by diminished social reciprocity and communication skills and the presence of stereotyped and restricted behaviours. Executive functioning deficits, such as working memory, are associated with core ASD symptoms. Working memory allows for temporary storage and manipulation of information and relies heavily on frontal-parietal networks of the brain. There are few reports on the neural correlates of working memory in youth with ASD. The current study identified the neural systems underlying verbal working memory capacity in youth with and without ASD using functional magnetic resonance imaging (fMRI).

**Methods:**

Fifty-seven youth, 27 with ASD and 30 sex- and age-matched typically developing (TD) controls (9–16 years), completed a one-back letter matching task (LMT) with four levels of difficulty (i.e. cognitive load) while fMRI data were recorded. Linear trend analyses were conducted to examine brain regions that were recruited as a function of increasing cognitive load.

**Results:**

We found similar behavioural performance on the LMT in terms of reaction times, but in the two higher load conditions, the ASD youth had lower accuracy than the TD group. Neural patterns of activations differed significantly between TD and ASD groups. In TD youth, areas classically used for working memory, including the lateral and medial frontal, as well as superior parietal brain regions, increased in activation with increasing task difficulty, while areas related to the default mode network (DMN) showed decreasing activation (i.e., deactivation). The youth with ASD did not appear to use this opposing cognitive processing system; they showed little recruitment of frontal and parietal regions across the load but did show similar modulation of the DMN.

**Conclusions:**

In a working memory task, where the load was manipulated without changing executive demands, TD youth showed increasing recruitment with increasing load of the classic fronto-parietal brain areas and decreasing involvement in default mode regions. In contrast, although they modulated the default mode network, youth with ASD did not show the modulation of increasing brain activation with increasing load, suggesting that they may be unable to manage increasing verbal information. Impaired verbal working memory in ASD would interfere with the youths’ success academically and socially. Thus, determining the nature of atypical neural processing could help establish or monitor working memory interventions for ASD.

## Background

Autism spectrum disorder (ASD) is a neurodevelopmental disorder characterised by diminished social reciprocity and communication skills, as well as the presence of stereotyped and restricted behaviours [1]. There is considerable evidence that individuals with ASD also have impaired executive and cognitive function [1–8]. The deficits in executive processing may contribute to the autistic symptomology, as proposed by the ‘executive dysfunction theory’ of ASD [5, 6]. Prior literature on the neural underpinnings of ASD, as well as the cognitive difficulties that follow, suggests that working memory (WM) impairments are associated with functional abnormalities in the frontal lobe, especially prefrontal cortical activity [3, 7, 9–12]. The protracted frontal lobe maturation means that the functions relying on the frontal lobes are particularly vulnerable to developmental disturbances [13, 14].

Working memory is the ability to temporarily store and manipulate information [15, 16]. WM is seen as an essential element of cognitive control [16–19], critical for learning and academic achievement [20], as well as social competency [21]. Previous literature suggests that individuals with ASD have greater difficulty with visuo-spatial than verbal WM, which is more often comparable to typically developing (TD) individuals [22–24]. Prior work also reports, however, that WM in ASD is intact for simple memory tasks [22–26] including simple verbal WM [27], but impaired on more complex tasks [22, 23, 25, 26, 28] including verbal WM [29], compared to typically developing (TD) individuals, or broadly compromised [30]. A number of studies found that when performing WM tasks of increasing complexity or cognitive load, children with ASD were impaired compared to TD children [8, 26, 29].

The neuroimaging literature has identified a system of lateral prefrontal, premotor and posterior parietal cortices underlying WM function [31, 32], with children showing more widespread activation patterns than adults [33]. During a verbal WM two-back task, Nagel et al. [34] found that children (ages 10–16 years) recruited the left frontal and temporal lobes. Similarly, Thomason et al. [35] used a verbal WM block design task and observed that children (ages 7–12 years) showed activation in the left frontal and parietal cortical regions, but activation in these regions was reduced compared to adults.

Few studies have used neuroimaging to investigate verbal WM in ASD, with most studies using visual-spatial tasks (e.g., [10, 12, 36, 37]); this, our understanding of the neural correlates underlying verbal WM deficits in ASD, particularly in children, remains modest. Koshino et al. [9] used a letter matching task and found that, despite comparable behavioural performance, adults with ASD showed right-lateralised activation in the dorsolateral prefrontal cortex (dlPFC) and parietal and inferior temporal areas, whereas TD adults showed bilateral dlPFC activation and less posterior activity. Following a multi-pronged analysis approach, the authors concluded that TD adults used verbal encoding strategies to complete the task, whereas adults with ASD used nonverbal and visually oriented strategies with their WM network shifted towards a right hemisphere dominance.

The ‘*n*-back’ protocol is commonly used to manipulate cognitive load while studying WM [9, 10, 27, 32, 38–45]. The typical *n*-back task involves viewing a series of stimuli, then indicating whether the current stimulus is the same as the one presented ‘*n*’ (1, 2, 3, etc.) trials before. The difficulty level is indexed by the total number of interfering items between repeating stimuli. By increasing load in this manner, different mental strategies required to complete the task are also employed, including executive functioning and procedural strategies. Manipulating both WM and other cognitive functions across load makes WM-specific changes difficult to quantify and link to specific brain regions. In the present study, we used a one-back letter matching task (LMT) [46–48] that avoids these confounds. LMT holds executive function constant across difficulty levels, while systematically manipulating memory load, which better isolates the effects of cognitive load on verbal WM. A developmental investigation of LMT in typically developing children and adults showed an opposing cognitive processing system, with increasing cognitive load and increasing recruitment of brain areas related to WM, while decreasing activation of areas in the default mode network (DMN); adults showed larger load-dependent changes than children in the bilateral superior parietal gyri, inferior/dorsolateral prefrontal and left middle frontal gyri [48].

Limited neuroimaging studies exist to examine the impact of WM load on brain activity in ASD. In a recent investigation by Rahko et al. [44], adolescents with ASD (ages 11–18 years) were observed to have reduced modulation of brain activity with increasing cognitive load in the insula, motor and auditory and somatosensory cortices compared to TD adolescents during a visuo-spatial *n*-back WM task. An earlier study by Vogan et al. [47] utilising a colour matching task (a visuo-spatial version of LMT) showed that children with ASD (ages 7–13 years) demonstrated reduced modulation in the dlPFC, medial premotor cortex and precuneus with increasing cognitive load.

The current study used functional magnetic resonance imaging (fMRI) with a verbal WM task to explore neural systems underlying WM, and the effects of cognitive load, in children and young adolescents with and without ASD. In this study, the cognitive load was manipulated by increasing task difficulty level (see the “Methods” section for full task description). We hypothesised that children with ASD would perform with a lower accuracy than their matched TD controls on the LMT with increasing cognitive load. Moreover, we expected that children with ASD would under-recruit frontal and parietal cortical regions related to verbal WM, relative to TD children, and that the difference would increase with greater cognitive demand. We predicted that cortical activity would be linearly modulated (increasing in WM areas, decreasing in DMN areas) by task difficulty; however, we anticipated that the youth with ASD would have a less pronounced pattern of linear activation/deactivation.

## Methods

### Participants

Ninety one participants (47 ASD, 44 TD) were recruited through community support centres, parent support groups, email listservs, hospital ads and schools for this study. Six TD participants and 20 ASD participants were excluded from analyses due to inadequate task performance; see below (lines 197–202) for our threshold for task performance (ASD = 12, TD = 2), protocol completion (ASD = 5, TD = 2) and excessive movement (ASD = 3, TD = 0), and two TDs were excluded for age-matching. The age- and sex-matched sample was composed of 27 children with ASD (5 girls and 22 boys) and 30 TD children (8 girls and 22 boys) aged 9 to 16 years old. Although groups differed slightly on IQ as determined by the Wechsler Abbreviated Scale of Intelligence [49], *t*_(39)_ = 2.16, *p* = 0.04, both groups had IQs within the average range, see Table 1 for additional participant characteristics.

**Table 1:** Demographic and neuropsychological test characteristics of the sample

Variables	ASD		TD		Significant test
	%	Mean (SD)	%	Mean (SD)	
Demographic data					
Sex (% male)	81		73		*χ*^2^_(1)_ = 0.54, *p* = 0.46
Age		12.56 (1.46)		12.96 (1.89)	*t*_(54)_ = 0.91, *p* = 0.37
Full-scale IQ		105.52 (14.41)		112.27 (7.91)	*t*_(39)_ = 2.16, *p* = 0.04*
ADOS total^a^		11.89 (4.30)		N/A	

*p < 0.05

^a^ADOS scores range from 3 to 20 with greater symptom severity reflected by higher scores

Participants were not included in the study with any significant psychiatric comorbidities [1], medical illnesses, neurological disorders, prematurity, colour blindness, uncorrected vision, IQ < 80 or any standard MRI contraindicators, such as ferromagnetic implants. TD participants were also not included if they had a history of learning disability, developmental delay, a sibling with ASD or attention deficit hyperactivity disorder (ADHD). These factors were not the current primary diagnosis for any of the ASD subjects.

Informed consent, MRI scanning, and the cognitive and clinical testing involved in this study were carried out at the Hospital for Sick Children in Toronto. All the experimental procedures used were approved by the hospital’s Research Ethics Board. All participants gave informed verbal assent, and a parent or legal guardian of all participants gave informed written consent.

ASD clinical diagnosis was confirmed through expert clinical judgement and the Autism Diagnostic Observation Schedule (ADOS) [50] for all participants with ASD. The ADOS was conducted by a trained individual with established inter-rater research reliability.

### Letter matching task

**Fig. 1:**
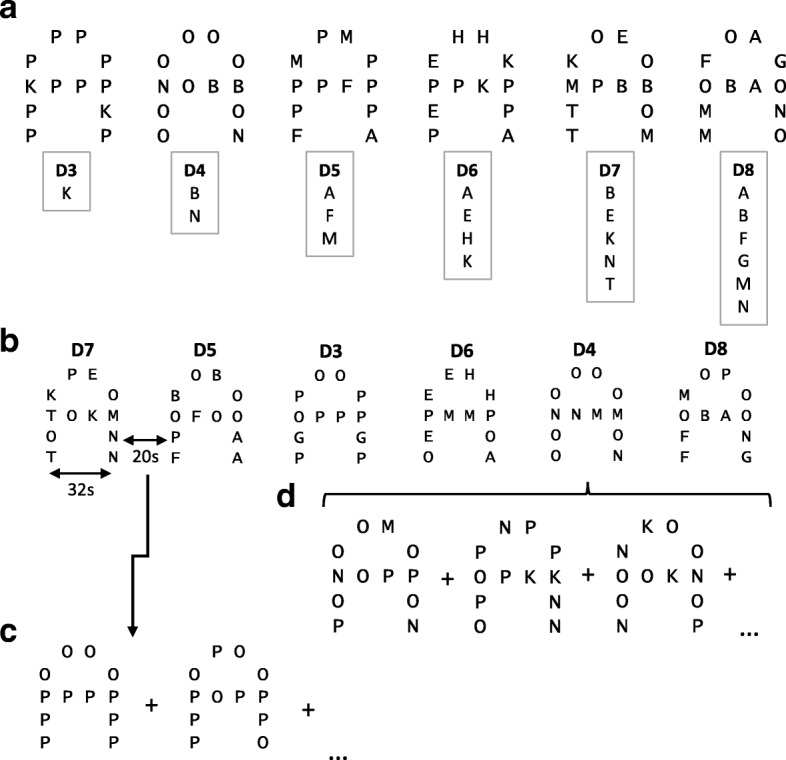
Protocol description of the letter matching task (LMT). **a** The task consisted of six difficulty levels where the number of relevant letters (A, B, E, H, K, M, N, T) increased with each difficulty level. Difficulty level = the number of relevant numbers + 2. Participants were instructed to ignore the global ‘A’ figure, letter location, letter repetition and irrelevant letters (‘O’ and ‘P’). **b** The task used a block design with each run consisting of 32-s task blocks for each difficulty level, followed by 20 s baseline blocks with figures containing only ‘O’ and ‘P’ (irrelevant letters). The task blocks were shown in pseudo-random order within each run. **c** An example of part of a baseline block sequence during which participants were instructed not to respond. **d** Example of part of a task block sequence; participants indicated if the current figure ‘A’ contained the same or different letters as the previous figure. In the first exemplar (in 1B), the target letters are M and N; in the subsequent exemplar (in 1D), the target letters are M and N (thus the same), then N and K (thus different) and then N and K again (thus, the correct response is ‘same’), as each stimulus is judged by whether the stimuli are the same or different as the preceding one. Stimuli were presented for 3 s followed by a 1-s inter-stimulus fixation cross

The LMT is a verbal WM task. LMT is presented visually to participants and has linguistic/phonological features. Participants attended to letters embedded in a global “A” figure. Participants were taught to focus only on the eight relevant letters (A, B, E, H, K, M, N, T) presented in uppercase and to ignore irrelevant letters “O” and “P” (Fig. 1). The task was designed with both relevant and irrelevant letters, as well as the irrelevant global letter, since tasks containing misleading or irrelevant features evoke interference and elicit cognitive control, which has been shown to provide more reliable measures of WM capacity [47, 51]. The number ‘*n*’ of relevant letters in the figure, referred to as capacity, increased by one item for each increasing difficulty level. Difficulty level was assigned *n* + 2 to account for these cognitive control and executive functions. LMT is a one-back task in which participants were instructed to identify relevant letter(s) and remember if the letter(s) in the current stimulus figure matched those from the previous figure, disregarding letter repetition and location. Repetition of both irrelevant and relevant letters within a stimulus was usual (see Fig. 1), and although the numbers and placement of the letters changed, the participants always ignored the same two letters, O and P. Stimuli were presented one at a time for 3 s, during which time children indicated their response using a dual-key MRI compatible keypad in their right hand; one button for the same relevant letters embedded in the stimulus as the previous stimulus and one button for different. A 1-s inter-stimulus interval during which a fixation cross was presented followed the task stimuli. The baseline trials included presentations in the same configuration as the task stimuli, except that the stimuli only included the irrelevant letters (O and P) in varying configurations. They were presented with the same timing as the task, but for 20 s; thus, only five stimuli per block (see Fig. 1c). All children were trained and completed practice trials successfully with an accuracy of at least 80% prior to performing the task in the scanner.

Twenty-four task and 24 baseline blocks (168 total task trials) were displayed over four runs. Each run included a 32-s block for each of the six difficulty levels; each task block consisted of eight stimuli of the same difficulty level. The levels were randomised for each run, with the same order of runs presented to all participants. The task blocks alternated with the 20-s baseline blocks, where participants were taught to look but not respond to the figures. Items were only correct if subjects responded correctly within 3 s of stimulus onset. The fMRI session took approximately 22 min, during which reaction time and accuracy were recorded as behavioural data.

Participants were excluded from the analyses if they did not complete at least three runs of the task, with an accuracy of at least 70% (averaged across their runs) on the two easiest levels (D3 and D4). Participants were also required to have at least two runs where at least 50% of the blocks were 70% accurate, to ensure that participants were performing better than chance (50%). Motion was considered acceptable if participants moved less than 1.5 mm from their average head position in a minimum of 60% of the volume within a task block.

### Image acquisition

All images were acquired on a 3T Siemens Trio MRI system with a 12-channel head coil. Foam padding was used to provide head motion restriction and stabilisation. fMRI scans were a single-shot echo planar imaging sequence (axial; FOV = 192 × 192 × 150 mm; 3 × 3 × 5 mm voxels; TR/TE/FA = 2000/30/70). The visual stimuli for the task (LMT) were shown using MR-compatible goggles. Stimuli were displayed, and performance was documented using presentation software (Neurobehavioral Systems Inc., Berkeley, CA, USA). Structural scans were used as anatomical references, collected as a high-resolution T1-weighted 3D MP-RAGE image (sagittal; FOV = 2000/30/70 mm; 1 mm iso voxels; TR/TE/TI/FA = 2300/2.96/900/9). During the structural scan, participants used MR-compatible goggles and earphones to watch a movie of their choice.

### Behavioural data analyses

Both TD and ASD groups performed poorly on difficulty levels 7 and 8 (D7 and D8), (TD: D7—*M* = 0.59, SD = 0.15; D8—*M* = 0.58, SD = 0.13; ASD: D7—*M* = 0.53, SD = 0.11; D8—*M* = 0.49, SD = 0.14). D7 and D8 were therefore excluded from the analyses, and the first four difficulty levels (D3 to D6) were analysed. Averages across runs for each group were generated for accuracy and response times at each difficulty level, which were analysed using two-way mixed ANOVAs with difficulty level (D3, D4, D5 and D6) as a within-subject factor and group (ASD and TD) as a between-subject factor.

### fMRI data analyses

fMRI data were preprocessed using tools from FMRIB’s Software Library: FSL [52] and AFNI [53]. The initial three volumes were discarded from each run to ensure scanner stabilisation. 3dvolreg was used for interleaved slice-timing and McFlirt motion correction; the data were smoothed in place using a 6-mm FWHM Gaussian kernel and temporally filtered (0.01–0.2 Hz) then converted to percent signal change from baseline volumes. Images were registered to the Montreal Neurological Institute (MNI) 152 brain template. The maximum Euclidean displacement (MD) travelled by any brain voxel was calculated for each volume from the six rigid body transformation parameters. This MD metric was used to identify volumes with motion surpassing the minimum motion threshold. Each subject’s average MD was used to examine group motion differences (TD: *M* = 0.47, SD = 0.40; ASD: *M* = 0.50, SD = 0.47; *t*_(51)_ = 0.25, ns.).

Data analyses were performed using FSL fMRI expert analysis tool (FEAT) [54]. The data were fit to a block-design general linear model combined with a gamma function used to model haemodynamic changes, with D3 to D6 task parameters. IQ and age were both assessed as confounding variables using FSL FEAT and were both found to have no significant impact on BOLD response during LMT. Linear trend analyses were performed using levels D3 to D6 with fixed-effects higher level modelling to examine areas that linearly modulated as a function of task difficulty. Linear trend analyses were chosen as this was the approach used in prior studies with the same type of working memory protocols [48, 55, 56]. Individual subjects’ results were averaged across runs, then between-group comparisons were conducted using FMRIB’s Local Analysis of Mixed Effects-1 (FLAME-1) [52]. Using FLAME-1 allowed us to acquire between-subject variance estimation, thus increasing our capacity to identify real activation [54]. Cluster-based thresholding was determined by *Z* > |2.3| as well as a corrected cluster significance threshold of *p*_corr_ < 0.05 to identify significant activations. Regions of interest (ROIs) were identified by examining local maxima of regions showing significant variation between TD and ASD groups in the linear trend analyses, for visualisation only. Spherical ROIs with 6-mm radii centred on the local maxima of cohort difference maps were created from which average percent signal change and standard error scores were derived. The average peak cluster signal change for both the TD and ASD groups was plotted as a function of difficulty to examine visually the verbal working memory activation patterns with increasing cognitive load.

## Results

### Behavioural data

**Fig. 2:**
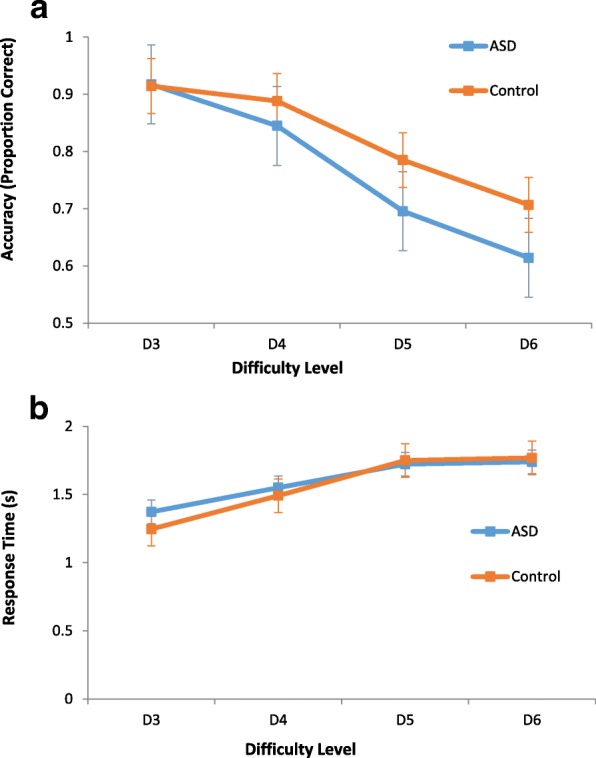
LMT behavioural performance. **a** Mean proportion correct for levels D3 to D6 with standard error bars. **b** Average reaction times for levels D3 to D6 with standard error bars

There was a weak but significant effect of group on accuracy, *F*(1,55) = 4.06, *p* = 0.049, in which TD children performed slightly better than children with ASD. There was a significant main effect of difficulty level on accuracy, with accuracy decreasing as a function of difficulty, *F*(2.50, 165) = 80.26, *p* < 0.001 (Greenhouse-Geisser corrected degrees of freedom). There was also a significant group × level interaction, *F*(2.50,165) = 2.98, *p* = 0.043 (Greenhouse-Geisser corrected), in which group differences in performance became larger with increasing task difficulty (see Fig. 2a). Post hoc *t* tests revealed that group performance did not differ on D3 (*t*(55) = 0.12, *p* = 0.91) and D4 (*t*(55) = 1.45, *p* = 0.15), whereas TD children performed somewhat better than children with ASD on D5 (*t*(55) = 2.15, *p* = 0.04) and D6 (*t*(55) = 2.14, *p* = 0.04).

There was no significant effect of group on response times, *F*(1,55) = 0.29, *p* = 0.59, or on group × level interaction effect, *F*(1.81, 165) =2.70, *p* = 0.077 (Greenhouse-Geisser corrected degrees of freedom). There was a significant effect of load on response times, *F*(1.81, 165) = 83.82, *p* < 0.001 (Greenhouse-Geisser corrected), with response times increasing as a function of difficulty across groups (see Fig. 2b).

### Within-group fMRI results

**Fig. 3:**
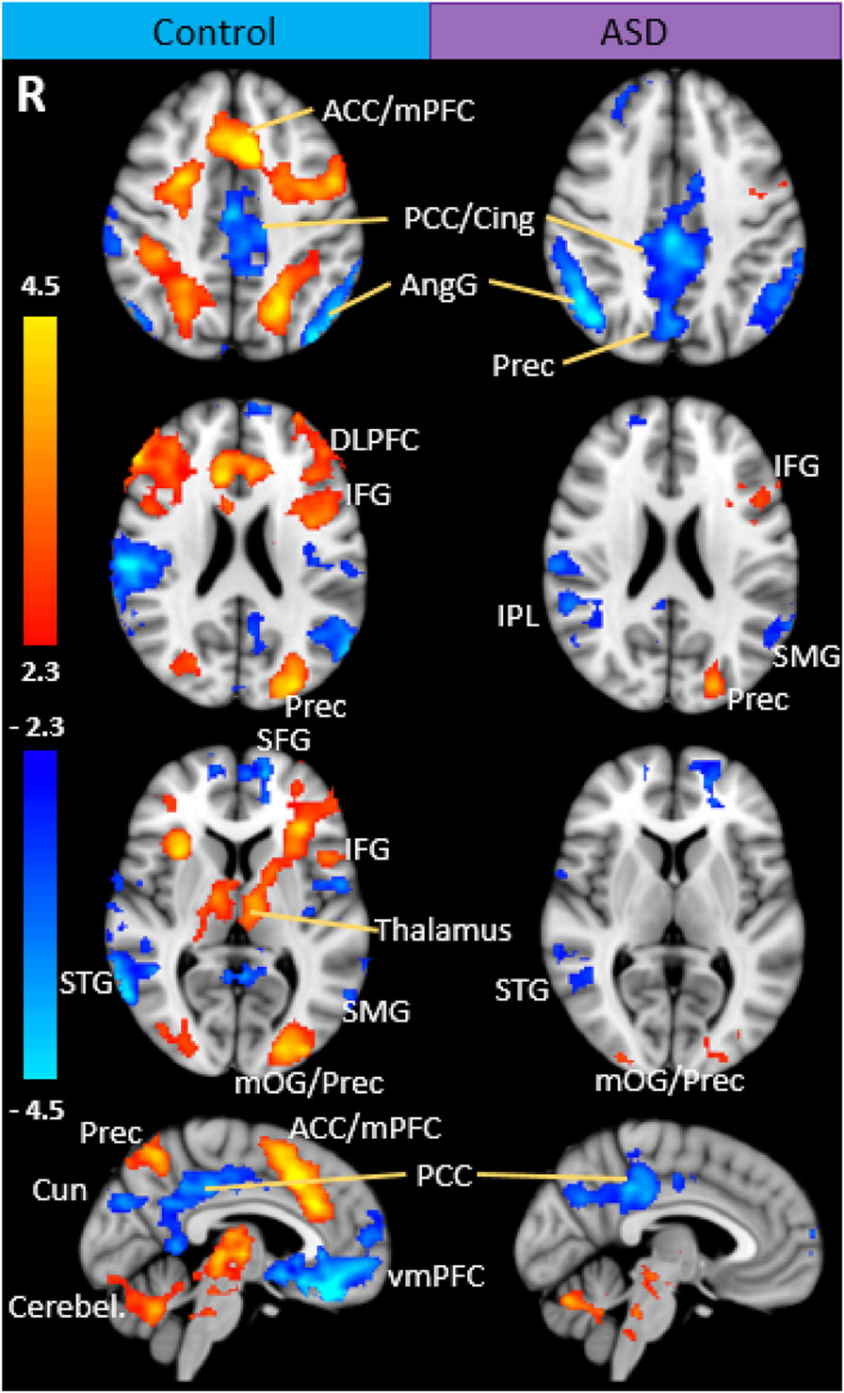
Group activation maps for the linear trend analyses in TD and ASD groups during LMT. Significant activations using cluster-based thresholding determined by *Z* > |2.3| and a corrected cluster significant threshold of *p* = 0.05. Areas in orange depict regions of increasing activation as a function of difficulty (positive linear relations between cortical activity and task difficulty level), and areas in blue depict regions of decreasing activation (negative linear relations between cortical activity and task difficulty level). *ACC* anterior cingulate cortex, *mPFC* medial prefrontal cortex, *PCC* posterior cingulate cortex, *Cing* cingulate, *AngG* angular gyrus, *Prec* precuneus, *dlPFC* dorsolateral prefrontal cortex, *IFG* inferior frontal gyrus, *IPL* inferior parietal gyrus, *SMG* superior medial gyrus, *SFG* superior frontal gyrus, *STG* superior temporal gyrus, *mOG* medial occipital gyrus, *Cun* cuneus, *Cereb* cerebellum, *vmPFC* ventromedial prefrontal cortex

Typically developing children showed significantly increasing activation as a function of increasing cognitive load (i.e. positive linear trend between BOLD signal and difficulty level) in the occipital, parietal, fusiform, cingulate and frontal areas (Fig. 3; see Table 2 for a complete list). Regions that showed decreasing activation as a function of load (i.e. negative linear relations between BOLD signal and difficulty level) included the medial frontal, anterior cingulate, bilateral temporal and parietal gyri and precuneus and cingulate cortices (Table 2 and Fig. 3).

**Table 2:** Linear trend analyses across difficulty levels for TD children

	Voxels	MNI coordinates			*Z* value	*p* value	Hem.	Region
		*x*	*y*	*z*				
Regions where activation increases with difficulty (increasing BOLD signal)	14,922	− 28	− 76	− 4	4.81	6.31 × 10^−25^	L	Middle occipital gyrus
	x	− 24	− 62	52	4.73		L	Superior parietal lobule
	x	− 26	− 72	− 10	4.51		L	Fusiform gyrus
	x	− 20	− 92	16	4.5		L	Cuneus
	x	28	− 58	58	4.44		R	Inferior parietal lobule
	14,033	− 8	18	42	5.41	6.92 × 10^−24^	L	Cingulate gyrus
	x	− 40	2	30	4.96		L	Inferior frontal gyrus
	x	10	22	38	4.83		R	Cingulate gyrus
	x	− 4	28	28	4.76		L	Anterior cingulate cortex
	3447	50	32	32	4.57	9.29 × 10^−9^	R	Middle frontal gyrus
	x	32	18	3	4.47		R	Insula
Regions where activation decreases with difficulty (decreasing BOLD signal)	5355	2	38	− 22	5.38	4.32 × 10^−12^	R	Medial frontal gyrus
	x	− 6	40	− 12	4.74		L	Medial frontal gyrus
	x	− 14	34	− 16	4.51		L	Inferior frontal gyrus
	x	6	62	− 6	4.5		R	Superior frontal gyrus
	x	2	14	− 6	4.41		R	Anterior cingulate cortex
	5165	− 60	− 26	− 18	4.72	8.85 × 10^−12^	L	Middle temporal gyrus
	x	− 40	− 78	40	4.54		L	Precuneus/angular gyrus
	x	− 56	− 62	30	4.46		L	Angular gyrus
	4472	60	− 22	20	4.84	1.31 × 10^−10^	R	Supramarginal gyrus
	x	66	− 30	34	4.56		R	Inferior parietal lobule
	x	60	− 56	2	4.45		R	Middle temporal gyrus
	x	68	− 38	12	4.24		R	Superior temporal gyrus
	3615	− 16	− 48	36	4.25	4.5 × 10^−9^	L	Cingulate gyrus
	x	2	− 44	34	4.04		R	Cingulate gyrus

Children with ASD showed increasing activation with greater WM load in the occipital gyri, fusiform, precuneus and inferior frontal gyrus (Fig. 3; see Table 3 for a complete list). Areas that showed decreasing activation across task difficulty included the parietal lobule, middle temporal, cingulate, precuneus and frontal gyri (Fig. 3 and Table 3).

**Table 3:** Linear trend analyses across difficulty levels for children with ASD

	Voxels	MNI coordinates			*Z* value	*p* value	Hem.	Region
		*x*	*y*	*z*				
Regions where activation increases with difficulty (increasing BOLD signal)	2170	− 28	− 76	− 6	4.25	3.64 × 10^−6^	L	Middle occipital gyrus
	x	− 24	− 68	− 6	4.09		L	Lingual gyrus/fusiform gyrus
	x	− 28	− 54	− 6	3.91		L	Lingual gyrus
	x	− 20	− 86	12	3.85		L	Cuneus/middle occipital gyrus
	x	− 22	− 86	22	3.82		L	Precuneus
	1120	− 2	− 74	− 26	4.19	1.31 × 10^−3^	L	Cerebellar vermis
	x	28	− 72	− 10	3.62		R	Fusiform/lingual gyrus
	x	0	− 58	− 32	3.51		L	Culmen/cerebellar vermis
	x	− 10	− 64	− 30	3.16		L	Cerebellum
	960	6	− 28	− 12	4.29	3.68 × 10^−3^	R	Thalamus
	x	6	− 34	− 20	4.01		R	Culmen
	x	− 8	− 24	− 12	3.43		L	Thalamus
	x	0	− 34	− 46	3.42		L	Brain-stem
	x	− 2	− 34	− 30	3.41		L	Culmen
	610	− 42	− 4	26	4.04	4.4 × 10^−2^	L	Inferior frontal gyrus
	x	− 58	18	32	3.89		L	Middle frontal gyrus
	x	− 38	22	18	3.05		L	Insula/inferior frontal gyrus
Regions where activation decreases with difficulty (decreasing BOLD signal)	3908	42	− 68	42	4.92	1.31 × 10^−9^	R	Inferior parietal/angular gyrus
	x	48	− 58	40	4.52		R	Inferior parietal lobule
	x	48	− 50	36	4.49		R	Angular gyrus
	x	48	− 46	36	4.46		R	Supramarginal gyrus
	x	46	− 44	0	4.1		R	Middle temporal gyrus
	3816	2	− 32	44	4.38	1.92 × 10^−9^	R	Cingulate gyrus
	x	− 6	− 38	38	4.19		L	Cingulate gyrus
	x	6	− 68	36	4.02		R	Precuneus
	x	12	− 46	30	3.79		R	Cingulate gyrus
	x	− 16	− 24	44	3.73		L	Cingulate gyrus
	1810	− 64	− 40	30	4.37	2.38 × 10^−5^	L	Inferior parietal lobule
	x	− 56	− 56	34	3.97		L	Angular gyrus
	843	− 12	62	12	4.07	8.14 × 10^−3^	L	Medial frontal gyrus
	x	− 22	66	14	3.77		L	Middle frontal gyrus
	x	− 18	52	8	3.58		L	Superior frontal gyrus
	x	16	58	4	3.33		R	Superior frontal gyrus
	x	− 20	42	4	3.32		L	anterior cingulate
	604	22	58	18	3.44	4.6 × 10^−2^	R	superior frontal gyrus
	x	24	52	32	3.29		R	middle frontal gyrus

Results from linear trend analyses from D3 to D6 for children with ASD. Areas that increased as a function of difficulty level are associated largely with visual processing, whereas areas that decreased as a function of difficulty level are associated with the default mode network. MNI coordinates represent the peak *Z* value of the cluster, *X* peak local maximas within cluster

### Between-group comparison

**Fig. 4:**
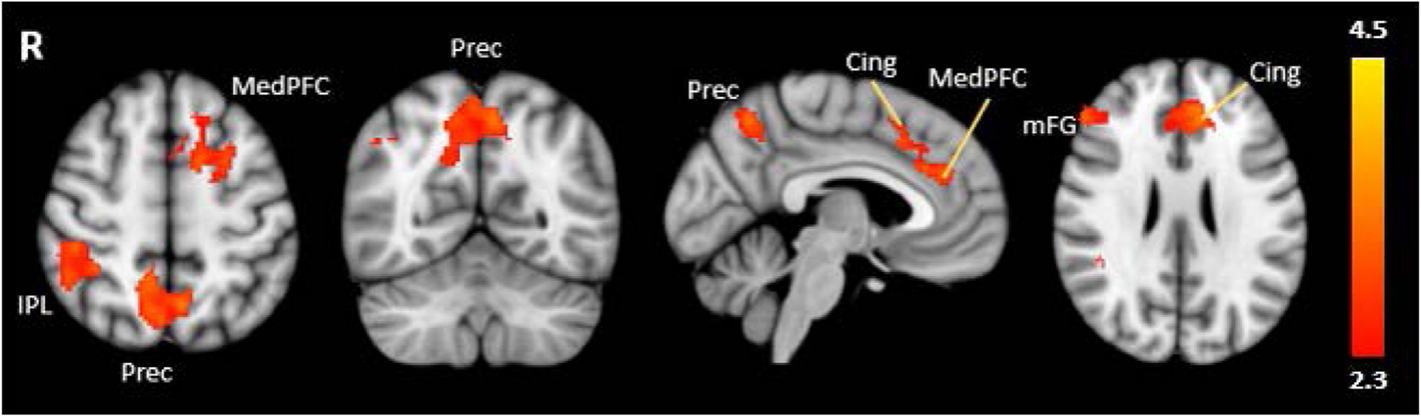
Results from between-group comparisons. Significant activations using cluster-based thresholding determined by *Z* > |2.3| and a corrected cluster significant threshold of *p* = 0.05. Areas in red/orange depict regions where the control children showed greater linear activation trends across difficulty level in the negative or positive direction than children with ASD. *medPFC* medial prefrontal cortex, *Cing* cingulate, *Prec* precuneus, *IPL* inferior parietal gyrus, *mFG* middle frontal gyrus

**Fig. 5:**
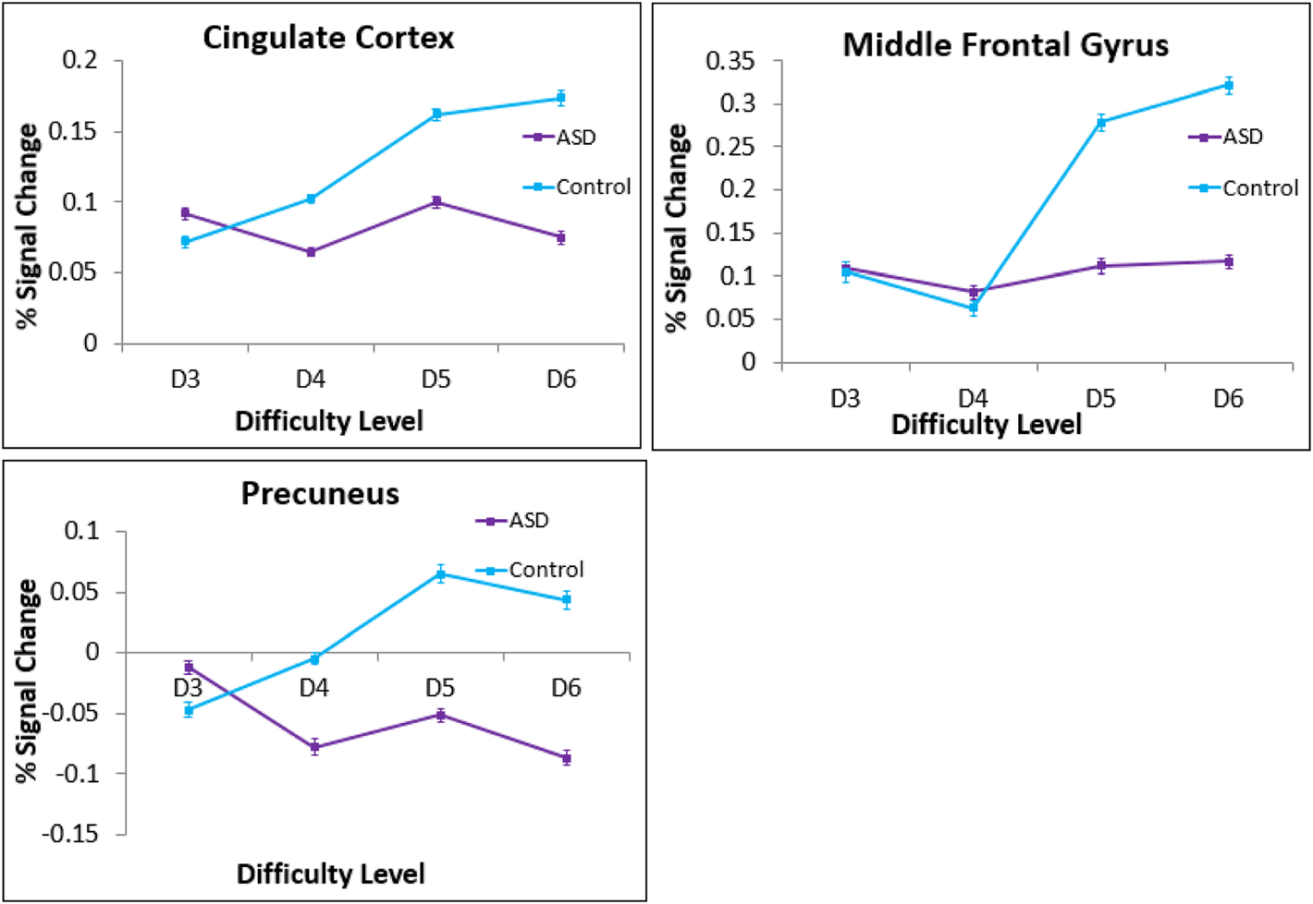
Mean peak cluster percent signal changes and standard errors plotted as a function of difficulty level. Areas where children with ASD differed significantly from TD children in the linear trend analyses

TD children had significantly stronger positive linear relations between activation and cognitive load compared to children with ASD in the bilateral prefrontal cortex, precuneus and inferior parietal lobule (Table 4; Fig. 4). In these regions, TD children showed increasing activation with increasing task difficulty, whereas the ASD group failed to show a positive linear trend (see Fig. 5 for graphs of the percent signal change of the ROIs of cortical areas that had significant between-group differences in linear patterns). There were no areas where the ASD group showed greater linear activation across task difficulty than TD children. There were no significant differences between groups in the patterns of deactivation with increasing cognitive load.

**Table 4:** Regions of significant differences between TD and ASD groups

Voxels	MNI coordinates			*Z* value	*p* value	Hem.	Region
	*x*	*y*	*z*				
1341	− 10	40	26	3.38	3.37 × 10^−4^	L	Medial frontal gyrus
x	− 14	8	50	3.32		L	Cingulate gyrus
x	− 16	6	62	3.26		L	Superior frontal gyrus
1175	38	− 36	44	3.58	9.25 × 10^−4^	R	Inferior parietal lobule
1095	14	− 52	46	3.7	1.53 × 10^−3^	R	Precuneus
x	− 6	− 62	52	3.32		L	Precuneus
833	54	36	22	3.73	8.73 × 10^−3^	R	Middle frontal gyrus
x	28	42	38	3.16		R	Superior frontal gyrus

Results from between group comparisons of the linear trend analyses from D3 to D6. All regions reported are areas where TD children showed greater positive linear relations between cortical activity and difficulty level (increasing BOLD signal with increasing task difficulty) than children with ASD. There were no areas where children with ASD showed greater linear relations between cortical activity and difficulty level than TD children. MNI coordinates represent the peak *Z* values of the cluster; *X* peak local maximas within cluster

## Discussion

Protracted development of the frontal lobes in combination with vulnerability to neurodevelopmental disturbances emphasises the need for deeper understanding of the function of these regions with typical and atypical development. Previous research has been centred on WM in adolescents and adults with ASD, leaving a gap in our understanding of WM in children with ASD. This is the first study to investigate the neural correlates of verbal WM in children and young adolescents with ASD compared to TD youth and examine the impact of cognitive load.

The TD group showed increasing recruitment of the brain areas classically linked to WM as a function of increasing cognitive demand and decreasing activation in regions associated with the DMN. The group with ASD, however, did not show this opposing system of cognitive processing. Specifically, TD children recruited the prefrontal and parietal cortical regions, areas directly correlated with verbal WM [3, 32, 38, 41, 42, 57], as a function of cognitive load significantly more than children with ASD who only demonstrated load-dependent deactivation in DMN regions. In a qualitative examination of activation patterns, we observed a larger spread of activation in children in this study compared to adults from Vogan et al.’s [48] study. This is consistent with the study by Geier et al. [33], who performed a visual spatial working memory oculomotor delayed-response task with adults, adolescents and children, and found that while all three age groups showed recruitment of a common network including the frontal, parietal and temporal regions, children and adolescents showed a wider distribution in addition to that network.

The behavioural data showed comparable performance on D3 and D4 between the TD and ASD groups; however, TD children performed with a higher accuracy on D5 and D6. These between-group differences emerging at higher cognitive loads are consistent with the literature suggesting that WM in children with ASD, when compared with TD children, is similar for simpler tasks but deficient for more complex tasks or those with greater cognitive demand [8, 22–25, 27, 29].

The ASD youth did not show comparable increasing activity in frontal-parietal regions with increased memory load, as the TD group. The frontal areas (BA 9) and inferior parietal lobe are classic areas for working memory [32], and activity in this WM task in the TD group was expected. The further activity that was greater in the TD group than the ASD group in the cingulate and precuneus could be due to increased recruitment of cognitive control mechanisms due to task difficulty, as both the anterior cingulate cortex (ACC) and precuneus are key hubs in cognitive networks. The differences between the groups were despite both completing the task successfully. Although the accuracy of the ASD group was lower than the TD group at the two higher load levels, they were performing the task similarly at D3 and D4 and were still at acceptable levels for D5 and D6. This suggests that the ASD group had unconventional utilisation of the brain areas for the WM task. This is concordant with the model that activation is more idiosyncratic in those with ASD, as reported elsewhere [58]. This leads to the usual regions not being seen in the ASD group analysis, and the more typical regions emerging as more active in the TD group in the group comparison. With a larger study, idiosyncratic patterns could be investigated specifically to determine if there are ASD subgroups with distinct alternative strategies.

Following on from this notion, future larger studies should also determine the role of other cognitive steps or strategies that may differ between TD and ASD groups that could influence WM performance. We included irrelevant aspects in the stimuli, to allow better determination of WM [51], but irrelevant details may also impact selective filtering and attention, which has been linked to working memory capacity [59]. As some researchers have found heightened visual processing in those with ASD [60, 61] particularly in relation to local features [62], this visual strategy may emerge more commonly in an ASD group and potentially impact strategy, and hence underlying neural recruitment. Future work could include assessments of visual processing skills (see [63]) and use that as a means of subgrouping participants by cognitive processing preferences.

A number of studies have reported atypical DMN activation in ASD [64–67], including an earlier investigation with the similar but visuo-spatial colour task (CMT) [55]. The DMN is a well-established network of the brain regions that are active during rest or non-task periods and show decreased BOLD signals during tasks [68], particularly tasks that are cognitively demanding. This modulation is believed to contribute to more efficient cognitive processing, and DMN regions are expected to deactivate with increasing task difficulty. The fact that here we saw no difference between the ASD and the TD groups in the decreasing activation of DMN regions with increasing task load could be due to a slightly older age range than previous studies, suggesting that DMN modulation may ‘catch up’ in children with ASD as they move into the teenage years. This is supported by a similar longitudinal protocol with somewhat older cohort [56], where the DMN modulation increased compared to 2 years earlier. These combined results indicate that the DMN modulation develops in ASD, albeit later than in the TD group, while the working memory processes remain distinct.

A limitation of the current study was the use of only linear models in the analyses. This was chosen as this is a subsequent study from our normative series [48], and a sister study to two other papers using a colour matching task [55, 56] all of which used the same analytic procedures, and we wanted to be able to relate the findings across the studies. Other approaches could be used in the future that investigate non-linear changes as a function of group (e.g. [69]) or with WM load, such as logarithmic changes that would be seen as rapidly increasing activation and then a plateau. Another limitation is that we had to exclude children who could not stay still in the scanner and who did not perform adequately on the task to ensure brain behaviour-related activation. By doing so, we were unable to include lower functioning children with ASD, and thus, our results are generalizable to higher functioning children only. There was also, on average, a lower IQ in the ASD youth and greater IQ variability. This is typical of this population, but even when IQ was covaried, the effects remained, suggesting that the effects were robust within the higher IQ range, despite the group differences in IQ. Further fMRI investigations are required with less demanding protocols to understand verbal WM function in low functioning children with ASD, who may present unique neural profiles. Finally, we had a wide age range in the study. We matched groups on age and age did not contribute to group effects. Nevertheless, smaller age ranges are ideal, and with a larger sample, age-related effects could be explored.

## Conclusions

The results from this study have several important implications. Our findings that children with ASD, relative to TD children, demonstrate inadequate modulation of neural capacity suggest that they could become overwhelmed with increasing verbal information. Impaired verbal working memory in ASD would have important academic and social implications. Specially, verbal WM difficulties could interfere with children’s ability to recall verbal information from conversations and social interactions, as well as to learn verbal material from classroom lessons or follow instructions. Determining the neural deficits of WM in children with ASD will help us understand the origins of the behaviours associated with ASD. Brain functional abnormalities in ASD may drive behavioural symptoms and give rise to cognitive impairments. Thus, exploring the neural correlates of WM contributes to knowledge of the ASD behavioural phenotypes. Finally, our study helps determine the nature of atypical neurodevelopment, which could help establish or monitor interventions for WM function in ASD.

## Abbreviations

ADOS: Autism Diagnostic Observation Schedule
ASD: Autism spectrum disorder
BOLD: Brain-oxygen-level dependent
D3, D4 etc.: Difficulty level 3, difficulty level 4, etc.
dlPFC: Dorsolateral prefrontal cortex
DMN: Default mode network
fMRI: Functional magnetic resonance imaging
FWHM: Full width half maximum
LMT: Letter matching task
MD: Maximum Euclidean displacement
MNI: Montreal Neurological Institute
PFC: Prefrontal cortex
ROIs: Regions of interest
TD: Typically developing
WM: Working memory
